# “Sniffing” out SARS-CoV-2 in Arizona working dogs: an exploratory serosurvey

**DOI:** 10.3389/fvets.2023.1166101

**Published:** 2023-05-05

**Authors:** Gavriella Hecht, Nathan Sarbo, Wayne Svoboda, Heather L. Mead, Irene Ruberto, John A. Altin, David M. Engelthaler, Heather Venkat, Hayley D. Yaglom

**Affiliations:** ^1^Arizona Department of Health Services, Phoenix, AZ, United States; ^2^Translational Genomics Research Institute, Flagstaff, AZ, United States; ^3^Hayden Road Animal Hospital, Scottsdale, AZ, United States; ^4^Centers for Disease Control and Prevention, Center for Preparedness and Response, Career Epidemiology Field Officer Program, Atlanta, GA, United States

**Keywords:** SARS-CoV-2, working dogs, neutralizing antibodies, surveillance, detection dog, police dog

## Abstract

Susceptibility to and infection with SARS-CoV-2 in companion animals has been well-documented throughout the COVID-19 pandemic. Surveillance for the virus in dogs has largely been focused on household pets; however, other canine populations may also be impacted. We partnered with a local veterinary hospital with a high working dog patient volume to conduct viral and neutralizing antibody testing in working dogs and identify potential risk factors in the dog’s work and home environments. Surveillance of SARS-CoV-2 in law enforcement and security working dogs in Arizona found 24.81% (32/129) of dogs to be seropositive. Thirteen dogs presenting with clinical signs or with reported exposure to COVID-19 in the 30 days prior to sample collection were also tested by PCR; all samples were negative. 90.7% (*n* = 117) of dogs were reported to be asymptomatic or have no change in performance at the time of sampling. Two dogs (1.6%) had suspected anosmia as reported by their handlers; one of which was seropositive. Known exposure to the dog’s COVID-19 positive handler or household member was identified as a significant risk factor. Demographics factors including sex, altered status, and type of work were not associated with canine seropositivity. Further work is warranted to understand the impact of SARS-CoV-2 and other infectious diseases in working dogs.

## Introduction

Working dogs play a critical role in supporting the health and safety of the public. These dogs are trained to perform specific tasks and are employed in meaningful work, including protection, detection, and therapy (1). Protection dogs are employed in roles including police patrol and hospital security, while detection dogs are trained specifically in narcotic or explosives detection, and search and rescue. Dogs are also involved in therapeutic roles such as crisis response. Working dogs often go through initial training with their handler, who is then responsible for their continued training (2). Though some live in agency-run kennels, many working dogs live with their handler and often interact with members of the household.

During the COVID-19 pandemic, susceptibility to and infection with SARS-CoV-2 was well-documented in companion animals, including dogs (3, 4). Evidence continues to show that the risk of companion animals spreading the virus to people is low, but that dogs can become exposed through close contact with infected people (5–7). Dogs can have asymptomatic SARS-CoV-2 infection or develop a range of respiratory and gastrointestinal signs. The full extent of SARS-CoV-2 clinical presentation in dogs is still unclear, including whether they experience a loss in their sense of smell, a hallmark sign of COVID-19 in people specifically associated with the Alpha and Delta variants (8). While scent detection abilities may not be as important for pet dogs, working dogs rely on this function to carry out their daily duties (e.g., detecting illicit substances or locating missing persons).

Surveillance for SARS-CoV-2 in dogs has largely been focused on household pets. While surveillance has been conducted for a variety of infectious diseases, such as bartonellosis and Chagas in working dogs (9, 10), efforts to understand SARS-CoV-2 in this population have been minimal. The main objectives of the study were to (1) conduct viral and neutralizing antibody testing in working dogs to determine how the SARS-CoV-2 virus impacts this population, and (2) identify potential risk factors associated with exposure to the virus during interactions in their work and home environments. Additional aims included identifying whether a change in overall performance, including scent detection was observed, in dogs exposed to SARS-CoV-2, and understanding the short and long-term implications.

## Methods

### Recruitment and sample collection

Dogs were recruited from a veterinary hospital in Maricopa County, Arizona, which experiences a high working dog patient volume. Approval for this work was granted by TGen’s Animal Care and Use Committee (#20163) and the ADHS’s Human Subjects Review Board (#20-0017). Written consent was received from the handlers of the dogs sampled.

Working dogs presenting to the veterinary hospital for routine examinations, vaccinations, or clinical illness were recruited by the veterinary hospital staff. Serum collection via venipuncture was performed on all dogs; these samples were initially centrifuged and stored in the refrigerator for 48–72 h. Working dogs with known exposure to COVID-19 within the past 30 days or dogs exhibiting signs compatible with viral infection also had nasal and rectal swabs collected. Swab samples were placed in conical vials with 2mls of sterile phosphate-buffered saline and frozen until shipment. A short questionnaire was administered to collect demographic information (e.g., dog breed, age, and sex), type of work the dog was involved in, COVID-19 exposure status, and any signs or symptoms observed by the handler.

### SARS-CoV-2 neutralizing antibody and viral testing

Serum was centrifuged at 2,500 rpm for 10 min upon arrival, aliquoted and tested for the presence of viral neutralizing antibodies (vNAbs) using the GenScript cPass™ SARS-CoV-2 Neutralizing Antibody assay per the manufacturer’s instructions (11). This is a competitive enzyme-linked immunosorbent assay (ELISA); presence of vNAbs will prevent the binding of the receptor-binding domain to the angiotensin-converting enzyme 2 coated wells, resulting in low optical density (OD). OD values were processed in R to produce inhibition (INH) percentage values (12). A sample was deemed positive if the INH value was ≥30% (11).

SARS-CoV-2 genomic material was extracted from nasal and rectal swab samples using Zymo DNA/RNA extraction kits. Extracted material was tested using a previously described real-time polymerase-chain reaction (rRT-PCR) assay (4, 13), developed and validated in-house, which targets the N and S protein of the virus. Samples were screened on the Bio-Rad CFX96 instrument after cDNA synthesis and denaturation; cycle threshold (Ct) values were generated to determine qualitative results. A sample was considered positive if Ct values for both targets were 38 or below.

Descriptive statistics were determined for breed, age, sex, altered status, type of work, health status, COVID-19 exposure status, SARS-CoV-2 serology, and PCR testing. Chi-square analysis was employed to assess the relationships between serology results and COVID-19 exposure status using SAS version 9.4.

Results were reported back to the veterinary hospital and the collaborating veterinarian communicated those to the handlers of the working dogs. If a working dog tested positive, recommendations regarding COVID-19 prevention strategies were provided to the handler. Handlers of seropositive dogs were also invited to participate in a semi-structured interview with project staff to gain a better understanding of canine medical history, household dynamics, and home and duty environments (14, 15).

## Results

During November 2021 through June 2022, 129 working dogs were recruited into the study. Serum samples were collected from all 129 enrolled dogs. Paired nasal and rectal swabs were collected on 13/129 dogs (10.1%) with known exposure to COVID-19 within the past 30 days or if the dog exhibited signs compatible with viral infection. Repeat sampling was performed on 4/13 dogs (3.1%) due to new exposures to COVID-19 positive people and to confirm serology results.

Table 1 summarizes the demographics of the working dogs enrolled. Dogs ranged in age from 9 months to 10 years (average 4.5 years). Belgian Malinois (*n* = 78) and German Shepherds (*n* = 15) were the most common breeds of dog, comprising 72.1% of the study population, although other breeds were represented. A majority of the dogs (*n* = 108) were intact males (63.6%) or spayed females (20.2%). Police patrol and detection was the most common role (43.4%, *n* = 56), followed by detection only (24.8%, *n* = 32), hospital patrol/security (16.3%, *n* = 21), and police patrol only (12.4%, *n* = 16).

**Table 1:** Characteristics and SARS-CoV-2 testing results of working dogs in Arizona (*N* = 129).

Characteristics/testing results	Number (%)
*Age*	
≤3 years	42 (32.6)
4–6 years	58 (44.9)
7–10 years	29 (22.5)
*Breed*	
Belgian Malinois (pure or mixed)	78 (60.5)
German Shepherd (pure or mixed)	15 (11.6)
Retriever (Labrador, Golden, or mixed)	21 (16.3)
Other (e.g., Bloodhound, Dutch Shepherd, Pointer)	15 (11.6)
*Sex/altered status*	
Intact male	82 (63.6)
Intact female	4 (3.1)
Male/neutered	17 (13.2)
Female/spayed *sex/altered status*	26 (20.1)
*Type of work*	
Police patrol (only)	16 (12.4)
Police patrol and detection	56 (43.4)
Detection* (only)	32 (24.8)
Hospital patrol/security**	21 (16.3)
Other (e.g., emotional support, crisis response)	4 (3.1)
*Health status of dog (at time of sampling or after handler’s COVID-19 infection)*	
Asymptomatic	117 (90.7)
Respiratory (e.g., coughing, difficulty breathing)	4 (3.1)
Gastrointestinal signs (e.g., vomiting, diarrhea)	3 (2.3)
Decreased scent detection	2 (1.6)
Other (e.g., general lethargy, decreased performance)	3 (2.3)
*Known exposure to SARS-Cov-2* ***	
Yes	69 (53.5)
No/unknown	60 (46.5)
*Evidence of SARS-CoV-2 vNAbs*	
Yes	32 (24.8)
No	97 (75.2)
*SARS-CoV-2 viral PCR detection*	
Yes	0 (0.0)
No	13 (10.1)
Not tested by *SARS-CoV-2 PCR*	116 (89.9)

* Includes search and rescue, narcotic, bomb, pest, and cadaver detection.

** Includes two explosive detection dogs in the hospital setting.

*** Includes exposure to a person with COVID-19 compatible symptoms or a person with a COVID-19 positive test.

90.7% (*n* = 117) of sampled dogs were reported to be asymptomatic or have no change in performance as noted by their handler at the time of sampling. Twelve dogs (9.3%) had mild clinical signs, including vomiting, diarrhea, coughing, lethargy, and decreased performance. Six of the 12 dogs were symptomatic at the time of sampling and 6/12 dogs had symptoms previous to collection but within the month after handler’s reported COVID-19 infection. Two of these dogs were diagnosed with Valley Fever. Of the 12 dogs, 1.6% (*n* = 2) were reported by their handlers to have “missed known finds,” indicating potentially compromised scent detection ability (i.e., suspected anosmia). Evidence of neutralizing antibodies was present in 24.8% (*n* = 32) of dogs; one (3.1%) of which had suspected anosmia. All 13 dogs tested by PCR was negative.

**Figure 1:**
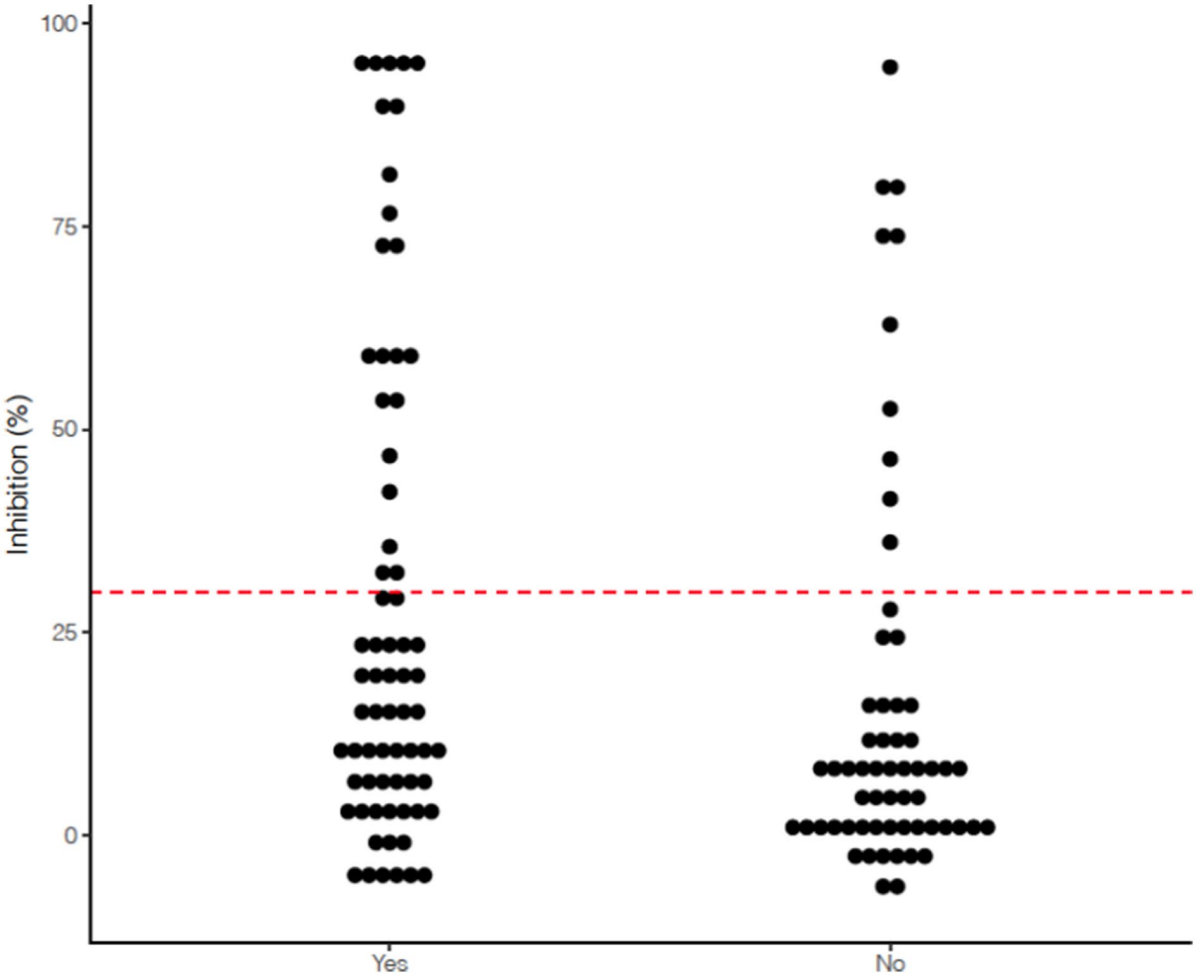
Scatterplot indicating spread of % inhibition of serum viral neutralizing antibodies measured using the GenScript cPass™ SARS-CoV-2 Neutralizing Antibody assay among 129 Arizona working dogs based on documented history of exposure to SARS-CoV-2 (Yes indicates known or suspected exposure to a person with COVID-19 compatible symptoms or a person with a COVID-19 positive test; No indicates no known or suspected exposure as reported by handler).

53.5% (69/129) of working dogs had known or suspected exposure to SARS-CoV-2 (e.g., handler had COVID-19), while 46.5% (60/129) had no documented exposures in their home or duty environment (Table 1). Of the 69 dogs with known exposure, 43 (62.3%) had an exposure within the 6 months prior to sampling (range 1–14 months, median 3–6 months between exposure and sampling). Assessment of exposure to SARS-CoV-2 specifically among the 32 seropositive dogs showed that 22 (68.8%) dogs had documented exposure, while 10 dogs (31.3%) did not. Working dogs with known or suspected exposure were more likely to test seropositive compared to those with no reported exposure based on chi-square analysis (68.8% vs. 31.3%, *χ*^2^ = 3.9525, *p* < 0.05), illustrated in Figure 1. Demographics factors including sex, age, altered status, and type of work were not associated with canine seropositivity. The timeframe between known SARS-CoV-2 exposure and sample collection date was also not found to be statistically significant.

Project staff conducted in-depth interviews with handlers for 40.6% (13/32) of seropositive dogs. All 13 dogs lived in the home of their handler; 92.3% were single family homes and 76.9% of homes had additional household members. Documented or suspected exposure to SARS-Cov-2 in the home environment was identified for all 13 (100%) dogs. It was further reported that these dogs, when off duty, had frequent and close interactions with their handler and other household members. While in a duty environment, 69.2% of dogs had regular interaction with their human co-workers (e.g., other law enforcement officers), but less than a quarter (23.1%) of dogs interacted with members of the public (e.g., during community events or in hospitals). Interviews also identified a recent history of valley fever in four (30.8%) dogs.

## Discussion

Working dogs bring diverse and critical skill sets to the law enforcement, military, and search and rescue environment, ultimately helping to protect the health and safety of the public (2). Given the nature of their work, these dogs are at risk of potential exposure to diseases and other health threats. Since the beginning of the pandemic, numerous studies have conducted SARS-CoV-2 surveillance in companion animals (3, 4, 6), however, to the authors knowledge, this is the first study to include a working dog cohort.

The authors originally hypothesized that working dogs may have a higher risk of SARS-CoV-2 exposure (and therefore likelihood of infection) due to their unique and expanded interactions in their duty environment compared to pet dogs. However, the results of this study are comparable with previous surveillance studies in pet dogs (3, 6, 13) and continue to support that close contact with people in a home or work environment (in this case COVID-19 positive handlers), are likely the main driver of SARS-CoV-2 exposure in dogs. The type of work and duty environment did not play a significant role in seropositivity of enrolled working dogs, which further disproves the original hypothesis. However, 31.3% (10/32) of dogs that were seropositive did not have a documented exposure. Of these 10 dogs, five were dual purpose (police patrol and detection), three were detention only, one did police patrol work only, and one was engaged in hospital security. This work highlights that working dog exposure to COVID-19 could occur unknowingly. Additionally, the true clinical impact and symptom manifestation, specifically loss of smell in dogs, has yet to be fully understood, given only two dogs enrolled in this study had reported anosmia.

There are a few noteworthy limitations to report. First, samples were collected from dogs as they visited the veterinary clinic for routine examination, vaccination, and only in some cases onset of clinical illness. The timing of sample collection, therefore, did not specifically correlate with possible SARS-CoV-2 exposure (although this data point was captured), and further explains why only a small number of enrolled dogs had swab samples collected. Second, the single-dilution SARS-CoV-2 neutralizing antibody assay used in this study does provide only a semi-quantitative measurement of antibody response and is not able to differentiate between a viral exposure that occurred more recently (e.g., 3 months) versus an exposure that occurred 6 months or more prior. Therefore, we were unable to pinpoint the exact timeframe of the dog’s exposure, unless clearly linked with known human positive cases in the household. An additional limitation is that complete information about potential exposures, work and home environment, and other risk factors were not captured for every dog enrolled. This limitation was recognized by the authors, and the study was designed to capture limited information from the canine handlers since the veterinary clinic was responsible for enrollment, consent, and sample collection. The goal was for the protocol to be minimally burdensome to the staff and fit into their standard operating procedures.

Exposure to varying endemic zoonotic diseases has been previously investigated in working dogs, and continuing this type of surveillance for emerging diseases, like SARS-CoV-2, in this population is important. Although only reported in a limited number of dogs, this study also sheds light on the potential for working dogs to diminish or lose their sense of smell after SARS-CoV-2 exposure. Further investigation in this area is needed to understand the impact that SARS-CoV-2 may have on this population of dogs’ work capacity. Agencies that deploy working dogs routinely assess scent detection capabilities during training, however these detection and mitigation plans may need revision. Additionally, this work can inform veterinary practices, policy development (e.g., mandatory SARS-CoV-2 vaccination for working dogs), and guide further management and prevention efforts that may be unique to this canine population (16).

## Data availability statement

The raw data supporting the conclusions of this article will be made available by the authors, without undue reservation.

## Ethics statement

The animal study was reviewed and approved by TGen’s Animal Care and Use Committee (#20163). Written informed consent was obtained from the owners for the participation of their animals in this study.

## Author contributions

GH, HY, and HV designed and coordinated the surveillance study with collaborating veterinary hospital. GH, NS, and HY led the preparation of the manuscript, including tables and figures. WS collected samples and data from dogs, as well as provided feedback on the manuscript. NS conducted the laboratory testing of the samples. IR provided guidance on analyses and reviewed manuscript. HM, JA, and DE provided technical support and oversaw the laboratory and surveillance efforts. Additionally, HM, JA, and DE provided important revisions to the manuscript. All authors contributed to the article and approved the submitted version.

## Funding

This study was supported in part by an appointment to the Applied Epidemiology Fellowship Program administered by the Council of State and Territorial Epidemiologists (CSTE) and funded by the Centers for Disease Control and Prevention (CDC) Cooperative Agreement Number 1NU38OT000297-03-00.

## Conflict of interest

The authors declare that the research was conducted in the absence of any commercial or financial relationships that could be construed as a potential conflict of interest.

## Publisher’s note

All claims expressed in this article are solely those of the authors and do not necessarily represent those of their affiliated organizations, or those of the publisher, the editors and the reviewers. Any product that may be evaluated in this article, or claim that may be made by its manufacturer, is not guaranteed or endorsed by the publisher.
